# Potential contribution of GST-T1 and GST-M1 polymorphisms in the onset of hepatic steatosis: from radiological to molecular and medico-legal analyses

**DOI:** 10.3389/fgstr.2024.1393282

**Published:** 2024-09-20

**Authors:** Vincenzo Cianci, Cristina Mondello, Gennaro Baldino, Giovanna Spatari, Angela Alibrandi, Alessio Cianci, Annalisa Cracò, Patrizia Gualniera, Alessio Asmundo, Michele Gaeta, Concetto Giorgianni, Daniela Sapienza

**Affiliations:** ^1^ Department of Biomedical and Dental Sciences and Morphofunctional Imaging, University of Messina, Messina, Italy; ^2^ Unit of Statistical and Mathematical Sciences, Department of Economics, University of Messina, Messina, Italy; ^3^ Department of Cardiovascular Medicine, Fondazione Policlinico Universitario A. Gemelli-IRCCS, Rome, Italy; ^4^ Department of Biomedical Sciences and Morphological and Functional Imaging, University Hospital Messina, Messina, Italy

**Keywords:** steatosis, NAFLD, *GSTs*, oxidative stress, magnetic resonance imaging, PDFF, forensic genetics, environmental exposure

## Abstract

**Introduction:**

Non-alcoholic fatty liver disease (NAFLD) is the most prevalent form of chronic liver disease in the world, and it is characterized by an excessive hepatic fat accumulation in more than 5% of hepatocytes documented by histology in the absence of alcohol consumption. It is a multifactorial pathology, where genetic component plays a fundamental role: the loss-of-function polymorphisms of genes coding for glutathione S-transferases would predispose to the pathology onset, also in the absence of other risk factors. The aim of the study was to evaluate the relation between the “NULL” GST-T1 and GST-M1 polymorphisms and the onset of NAFLD.

**Methods:**

A group of 117 “apparently healthy” Caucasian volunteers, selected from a larger population through the analysis of previously administered short questionnaires, underwent both magnetic resonance imaging-proton density fat fraction (MRI-PDFF) and buccal swabs: the aim was to identify the possible presence of hepatic steatosis and of the aforementioned “NULL” polymorphisms of interest.

**Results:**

A statistically significant association between the *GST-T1* and *GST-M1* “NULL” genotypes and the probability of developing NAFLD has been identified. In particular, the *GST-T1* “NULL” genotype has been associated with a greater probability of developing steatosis in early age, while the *GST-M1* “NULL” genotype seems to increase the risk of developing a higher grade of steatosis. No statistically significant correlations between the “NULL” genotype and sex have been detected.

**Discussion:**

Among the numerous risk factors capable of predisposing to NAFLD onset and progression, the genetic factors seem to play an important role. In particular, *GST-T1* and *GST-M1* “NULL” polymorphisms would appear to acquire even greater importance, as their loss of function results in an increase of oxidative stress. At high concentrations, ROS can determine oxidative modifications of cellular macromolecules, such as lipids, determining their accumulation into hepatocytes. The study also highlighted the importance of MRI-PDFF for hepatic steatosis diagnosis: this method allows the acquisition of data comparable to those of conventional biopsy; however, it permits the entire liver parenchyma to be visualized.

**Conclusion:**

A statistically significant correlation between the presence of *GST-T1* and *GST-M1* “NULL” genotypes and the presence of hepatic steatosis has been found.

## 1 Introduction

“The future is fatty”: so begins a 2013 editorial aimed at clarifying the challenges for an early diagnosis (lifestyle, genetic, imaging) of non-alcoholic fatty liver disease (NAFLD) (1). NAFLD is the most prevalent form of chronic liver disease in the world, frequently associated with obesity, type 2 diabetes mellitus, and insulin resistance (IR) (2).

NAFLD is characterized by an excessive hepatic fat accumulation, often responsible for the onset of IR, and it is defined by the presence of steatosis in >5% of hepatocytes (according to histological analysis), without ethanol consumption. In fact, the diagnosis of NAFLD requires the exclusion of both secondary causes and a daily alcohol consumption greater than P30 g for men and P20 g for women (3).

NAFLD is considered a multifactorial disease, a product of the numerous interactions between environmental factors, nutrition, and genetic factors. The interaction between all these factors is responsible for the disease phenotype and its progression (4, 5).

Given the growing interest of the scientific community on the genetic basis of NAFLD, in recent years, various associations have been described at the genomic level, particularly on some genes, which have improved our knowledge on the genetic basis of NAFLD (5). In fact, it seems that some genetic polymorphisms increase an individual’s susceptibility to environmental factors that, consequently, lead to the development of NAFLD, even in the case of an apparent lack of other risk factors (5). However, recently, the American Association for the Study of Liver Diseases (AASLD) in collaboration with other associations suggested a new nomenclature for steatotic liver disease, particularly the use of metabolic dysfunction-associated steatotic liver disease (MASLD) in place of NAFLD (6). This change has been introduced because several doubts were highlighted regarding the appropriateness of the term “non-alcoholic” that did not express accurately the etiology of the disease (for example, it is well known that there are overlapping biological processes that contribute to both NAFLD and ALD) (6). Moreover, the term “fatty” has been described as stigmatizing (6). Thus, the nomenclature MASLD, defined as the presence of hepatic steatosis associated with one cardiometabolic risk factor and no other discernible cause, has been widely accepted (6).

Since the first genome-wide association study (GWAS) was conducted to evaluate the genetic susceptibility of NAFLD, numerous other candidate gene studies have been conducted (7, 8).

The scientific literature has proposed several genes as both potentially responsible for the onset of this pathology and capable of influencing its natural history: among these, the membrane-bound O-acyl-transferase domain-containing 7 (MBOAT7), patatin-like phospholipase domain-containing 3 (PNPLA3), transmembrane 6 superfamily member 2 (TM6SF2), 17-beta hydroxysteroid dehydrogenase 13 (HSD17B13), and glucokinase regulator (GCKR) would appear to be those of greater interest (9). Further studies have focused on the possible association between increased oxidative stress and the onset of NAFLD (10).

In fact, there is a growing line of evidence that polymorphisms in genes encoding enzymes responsible for xenobiotic metabolism are among the key players in determining interindividual susceptibility to liver-related diseases (6). Oxidative stress and reactive oxygen species (ROS) production have also been widely implicated in the development of NAFLD. ROS production promotes lipid peroxidation, which leads to the formation of extremely reactive aldehyde compounds and the consequent damage at the intracellular level. At the same time, a decrease in antioxidant compounds is observed, such as *GSTs*, superoxide dismutase, and catalases (6).

Therefore, it can be stated that fatty liver is due to an interplay between genetic and environmental factors, with a major role played by oxidative stress.

In oxidative stress, many xenobiotic agents are metabolized by enzymes of both “phase I,” leading to the formation of procarcinogenic substances through oxidative reactions, and “phase II,” where metabolic intermediate products of the oxidative process are conjugated with endogenous mediators, leading to the production of hydrophilic products that can be easily excreted by the organism. *GST* is an example of a heterogeneous group of “phase II” enzymes (10).

*GSTs* are ubiquitous enzymes that catalyze the conjugation of reduced glutathione to electrophilic centers of various exogenous and endogenous substrates (xenobiotics, drugs, poisons, and ROS). Their importance in the human antioxidant system is confirmed by the significantly increased risk of oxidative stress‐related diseases in subjects bearing a “NULL” genotype for specific *GST* isoforms (i.e., homozygosity for genetic deletion), resulting in the lack of phenotypic expression of the corresponding enzyme (11, 12).

In humans, seven different isoforms of this enzyme have been found (α, ξ, μ, π, θ, σ, ω). Among these, *GST-M1* falls into the μ class, while and *GST-T1* falls into the θ class (13).

It is known that these genes can be affected by several mutations, capable of determining either a functional reduction or a complete absence of the related protein (14).

Numerous studies have also demonstrated that the functional alteration of the proteins encoded by the aforementioned genes renders their detoxification capacity ineffective, increasing oxidative stress (15). In fact, the “NULL” genotypes of *GST-M1* and *GST-T1* have been associated with an increased risk of developing several pathologies, including NAFLD, type 2 diabetes, and drug-induced hepatotoxicity (15).

Furthermore, literature data indicate geographic and ethnic variations in the frequency distribution of the “NULL” genotypes (12). Recent studies have documented that the percentage of individuals who do not express the enzyme encoded by the *GST-M1* polymorphism is significantly higher in Caucasian and Asian populations than in the African population, while the frequency of the “NULL” genotype of the GST-T1 would be in the order of 60% among Asians, 40% among Africans, and approximately 20% among Caucasians (12, 16–18). The detected high frequency of the “NULL” genotype of these genetic polymorphisms (more than 50% for the *GST-M1* polymorphism) has also been confirmed by a population study conducted in Eastern Sicily (11, 19).

The study aims to evaluate the relation between the *GST-T1* and *GST-M1* gene polymorphisms and the presence of NAFLD and to confirm the importance of magnetic resonance imaging-proton density fat fraction (MRI-PDFF) in quantifying liver fat fraction. MRI is considered a “state-of-the-art weapon” to quantify liver steatosis, considering that its reliability is by far better than ultrasonography, being comparable to biopsy.

## 2 Methods

A group of 117 volunteers were selected from a larger population through the analysis of previously administered short questionnaires: these questionnaires had the aim of selecting “apparently healthy” subjects, i.e., apparently without any risk factors.

In fact, the questionnaire was aimed at obtaining information on eating habits, smoking, lifestyle, professional profile (near and remote pathological history, physiological history, work history), use of drugs, and/or presence of concomitant pathologies, including those with metabolic disorders, ascertained by negative blood tests performed within the previous 3 months. Therefore, all enrolled subjects have a body mass index (BMI) between 18.5 and 24.9 and declare that they do not take drugs for any pathologies. The questionnaire was administered to Caucasian individuals residing in northeastern Sicily.

Both authorizations for the processing of sensitive data and informed consent have been acquired. These subjects then underwent genetic analysis.

### 2.1 Genetic research

Each patient underwent sampling of buccal mucosa cells through sterile tamponade. DNA extraction was carried out according to the Chelex^®^ 100 method, starting from the individual buccal swabs belonging to each subject of the group under study. Oligonucleotide sequences reported in the literature were used as the primer for the polymerase chain reaction (PCR) (16).

A multiplex has been set up, which is useful for the co-amplification of the loci *GST-T1*, *GST-M1*, and β-globin (the latter as a positive control of the reaction itself), in a final volume of 25 μl including 2.5 μl of extract (5–250 ng of DNA), 0.5 μM of each primer, 2.5 μl of Taq buffer (10× PCR Buffer II, Applied Biosystems, Waltham, Massachusetts, USA), 2 μl of MgCl 25 mM (Applied Biosystems, Waltham, Massachusetts, USA), 0.5 μl of dNTP mix (10 mM of PCR Nucleotide Mix, Promega, Madison, Wisconsin, USA), and 1 U of Taq polymerase (DyNAzyme II DNA polymerase, Finnzymes, Espoo, Finland).

The reaction was carried out with the use of a thermocycler (PCR Sprint, Hybaid, Teddington, United Kingdom) for a total of 30 amplification cycles: denaturation at 95°C for 1 min, annealing at 60°C for 1 min, and extension to 72°C for 1 min. The analysis of PCR products was executed with vertical electrophoresis on denaturing gel 6% polyacrylamide in ultrathin 7 M urea (0.4 mm) in TBE buffer 1×, using the following running conditions: 2,000 V, max mA, max W, for 150 min.

Migration bands related to the amplification products (480 bp for *GST-T1* and 215 bp for *GST-M1*) were visualized by silver staining and identified by comparing the number of base pairs to a standard DNA ladder (DNA pGEM^®^ marker, Promega, Madison, Wisconsin, USA). The following primers were used:

- GST-M1:

• forward primer 5′-GAACTCCCTGAAAAGCTAAAGC-3′,
• reverse primer 5′-GTTGGGCTCAAATATACGGTGG-3′;

- GST-T1:

• forward primer 5′-TTCCTTACTGGTCCTCACATCTC-3′,
• reverse primer 5′-TCACCGGATCATGGCCAGCA-3′.

### 2.2 Magnetic resonance imaging

Fat quantification (PDFF) was performed with a 1.5-Tesla scanner using a GE Quant T1 3D multi-echo sequence with the following parameters:

- repetition time (TR) 500 ms;
- six echoes with first echo time (ET) 1.5 ms and interecho spacing 1.3 ms;
- flip angle 5°;
- acquisition time 13 s.

Nowadays, hepatic PDFF is a standardized technique based on MRI. It represents a reproducible biomarker of liver fat content that has been applied as an endpoint in clinical trials and whose successful implementation into clinical care is an important goal. On the other hand, MR spectroscopy (MRS), an accepted non-invasive reference standard for PDFF measurement, is not widely available. Moreover, it is capable of sampling only small portions of the liver, leading to sampling variability. However, it allows an accurate *in-vivo* differentiation of the characteristic fat peaks which cannot be observed with PDFF (17).

MRI fat quantification methods use a low flip angle to minimize the spin-lattice (T1*) relaxation time, acquire multiple echoes to measure the difference in the characteristic relaxation for water and fat species when they relax in-phase and out-of-phase, and incorporate into their mathematical model the multifrequency interference effects of protons in fat (18). Several studies in adults have shown that MRI-estimated PDFF (MRI-PDFF) agrees closely with MRS-measured PDFF (MRS-PDFF) and biopsy, thus suggesting their future wide use for the quantification of hepatic PDFF in populations instead of MRS or biopsy (19).

According to the literature, steatosis value made it possible to divide the enrolled subjects into four classes: healthy individuals (steatosis value less than 5%), individuals with mild steatosis (6%–33%), individuals with moderate steatosis (34%–66%), and individuals with severe steatosis (>66%).

### 2.3 Statistical analysis

The numerical data were expressed as mean, standard deviation, median, and range (minimum and maximum), and the categorical variables as absolute frequencies and percentages.

The non-parametric approach was used since numerical variables were not normally distributed, verified by the Kolmogorov–Smirnov test.

The Spearman correlation test was applied in order to assess the possible correlation between the percentage of steatosis, age, and null positivity of genotypes T1 and M1.

In order to assess the existence of significant differences between categories of genotype (NULL vs. NO NULL), the Mann–Whitney test was applied with references to numerical parameters (age, percentage of steatosis), and the chi-square test was used for categorical variables (gender and grade of steatosis). Some boxplots were realized to better visualize the data.

Finally, a cumulative proportional odds model was estimated in order to identify significant predictive factors of the grade of steatosis, since response variables were ordinal on three ordered levels; the covariates were gender, age, *GST-T1*, and *GST-M1*. These models can be implemented through the Polytomous Universal Model (PLUM) procedure of the statistical software SPSS (Norušis, 2009).

Statistical analyses were performed using SPSS 27.0 for Windows package. A *p*-value lower than 0.05 was statistically significant.

## 3 Results

A population of 117 apparently healthy volunteers underwent both genetic test for the identification of *GST-T1* and *GST-M1* gene polymorphisms and MRI exams to evaluate the presence and grade of NAFLD. The subjects were between 27 and 69 years of age, with an average age of 49.98, consisting of 61 men (52.1%) and 56 women (47.9%).

**Figure 1:**
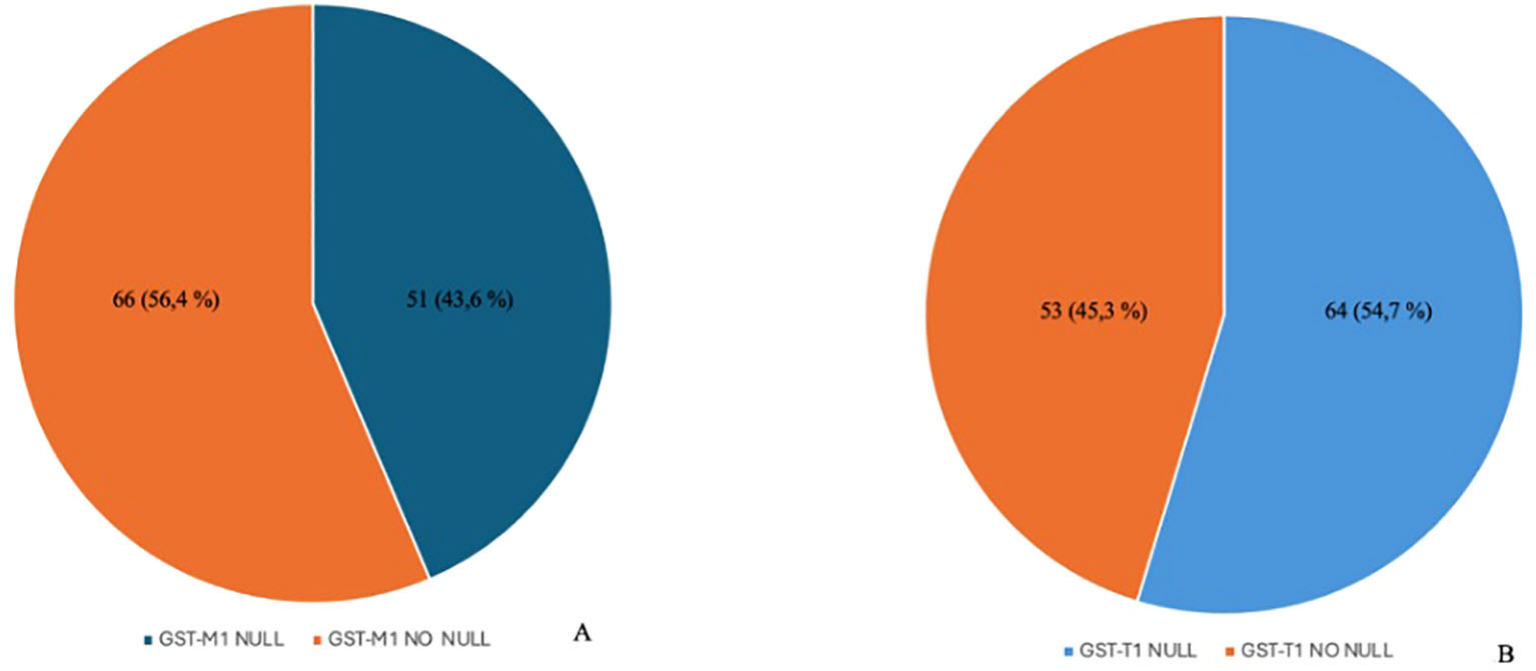
Distribution of polymorphisms in the enrolled population. The image shows a higher prevalence of the GST-M1 NO NULL genotype compared to the NULL genotype **(A)** and the prevalence of the GST-T1 NULL genotype compared to the NO NULL genotype **(B)**. GST-M1, Glutathione S-transferases Mu 1; GST-T1, Glutathione S-Transferase Theta 1.

After performing genetic analysis, the following results were obtained: 53 subjects (45.3%) had “NO NULL” *GST-T1* polymorphism and 64 (54.7%) had “NULL” *GST-T1* polymorphism. Sixty-six subjects (56.4%) had “NO NULL” *GST-M1* polymorphism, and 51 (43.6%) had *GST-M1* polymorphism (**Figures 1A, B**).

**Figure 2:**
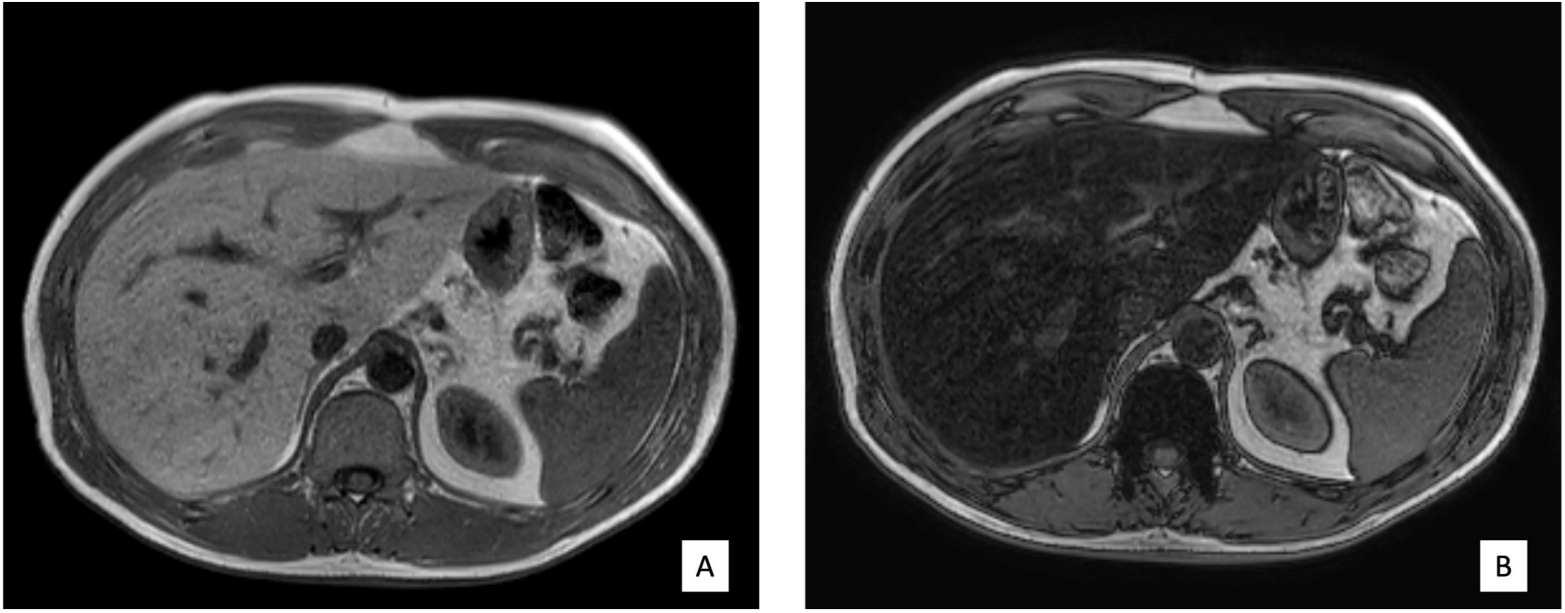
**(A, B)** Case 32: in-phase images showing loss of hepatic signal on opposed-phase images, demonstrating the presence of steatosis.

**Figure 3:**
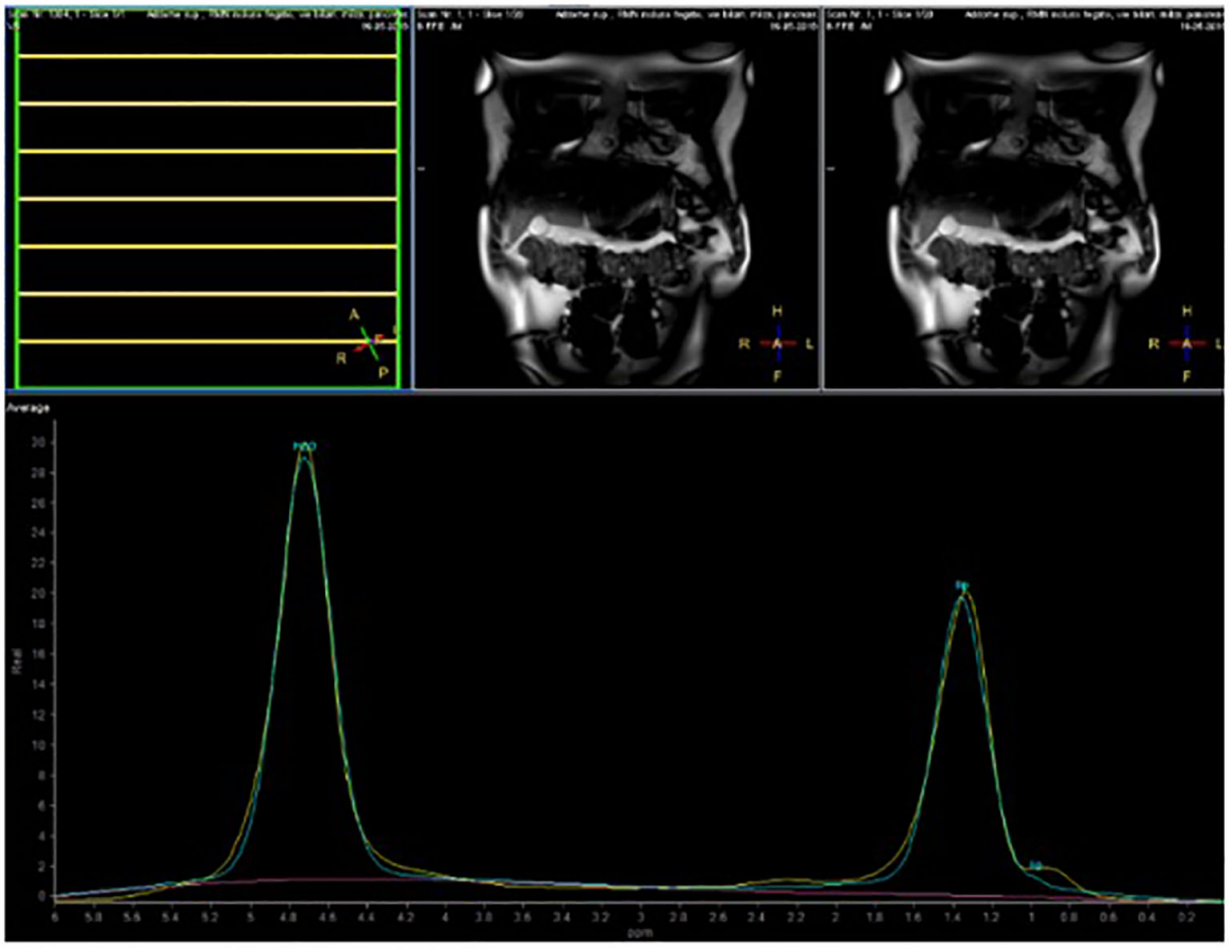
Case 32: spectroscopy evaluation.

**Figure 4:**
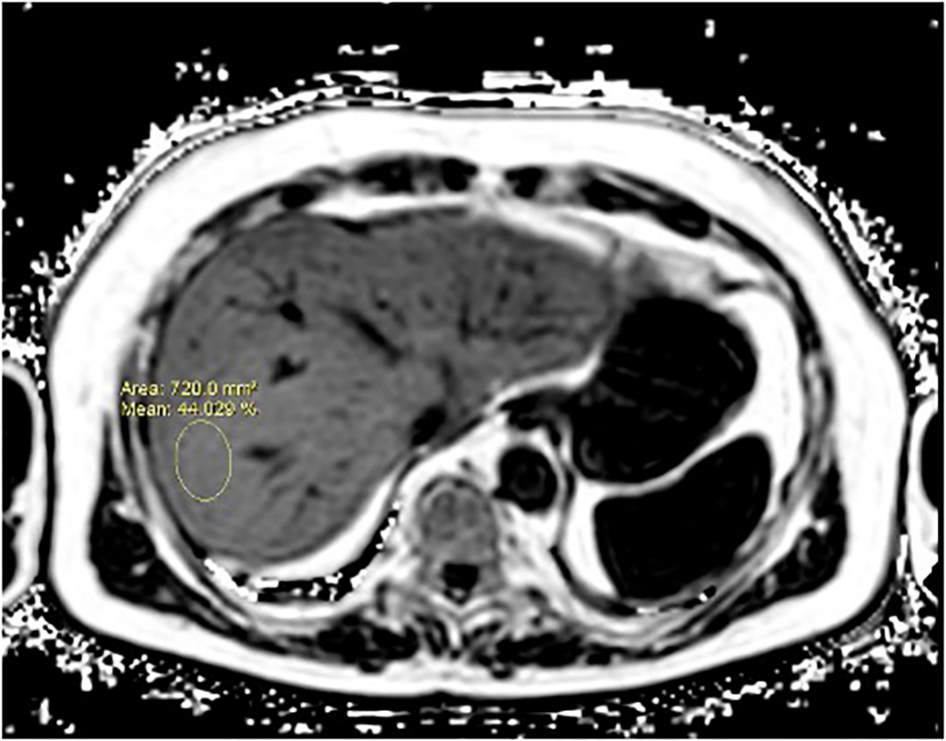
A case of NAFLD evaluated with PDFF. PDFF offers a precise, non-invasive quantification of liver fat content, covering the entire organ and providing consistent, reproducible results. It also correlates well with histopathological findings, making it a reliable alternative to biopsy for comprehensive liver fat evaluation.

The volunteers subsequently underwent MRI: 28 subjects (23.9%) had no steatosis (steatosis percentage <5%), 64 (54.7%) had mild steatosis (steatosis percentage 6%–33%), and 25 (21.4%) had moderate steatosis (steatosis percentage 34%–66%). No subjects with severe steatosis (>66%) were identified (**Table 1**). GE T1-weighted in-phase images show loss of hepatic signal on opposed phase images, demonstrating the presence of steatosis (**Figures 2A, B**). However, a quantification of fat fraction is not possible. On the other hand, both spectroscopy and PDFF sequence allow a reliable evaluation of the liver fat fraction. The PDFF sequence allows to evaluate the whole liver (**Figures 3**, **4**).

**Table 1:** Absolute frequencies and percentages for genetic parameters.

5.1.1 GST-T1		
	Frequency	%
NO NULL	53	45.3
NULL	64	54.7
Total	117	100.0
GST-M1		
	Frequency	%
NO NULL	66	56.4
NULL	51	43.6
Total	117	100.0
5.1.2 MRI		
	Frequency	%
No steatosis	28	23.9
Mild steatosis	64	54.7
Moderate steatosis	25	21.4
Total	117	100.0

The association between the two polymorphism types detected was subsequently evaluated: 33 subjects (28.21%) had a “NULL” genotype for both *GST-T1* and *GST-M1*, 33 (28.21%) had a “NULL” genotype for *GST-T1* and “NO NULL” for *GST-M1*, 19 (16.24%) had a “NO NULL” genotype for *GST-T1* and “NULL” for *GST-M1*, and 35 (29.91%) had a “NO NULL” genotype for both *GST-T1* and *GST-M1* (**Table 2**).

**Table 2:** Association between polymorphism types.

		GST_T1		
		NULL	NO NULL	Total
GST-M1	NULL	34 (28.21%)	19 (16.24%)	53 (45.3%)
	NO NULL	32 (28.21%)	32 (29.91%)	64 (54.7%)
	Total	66	51	117

**Figure 5:**
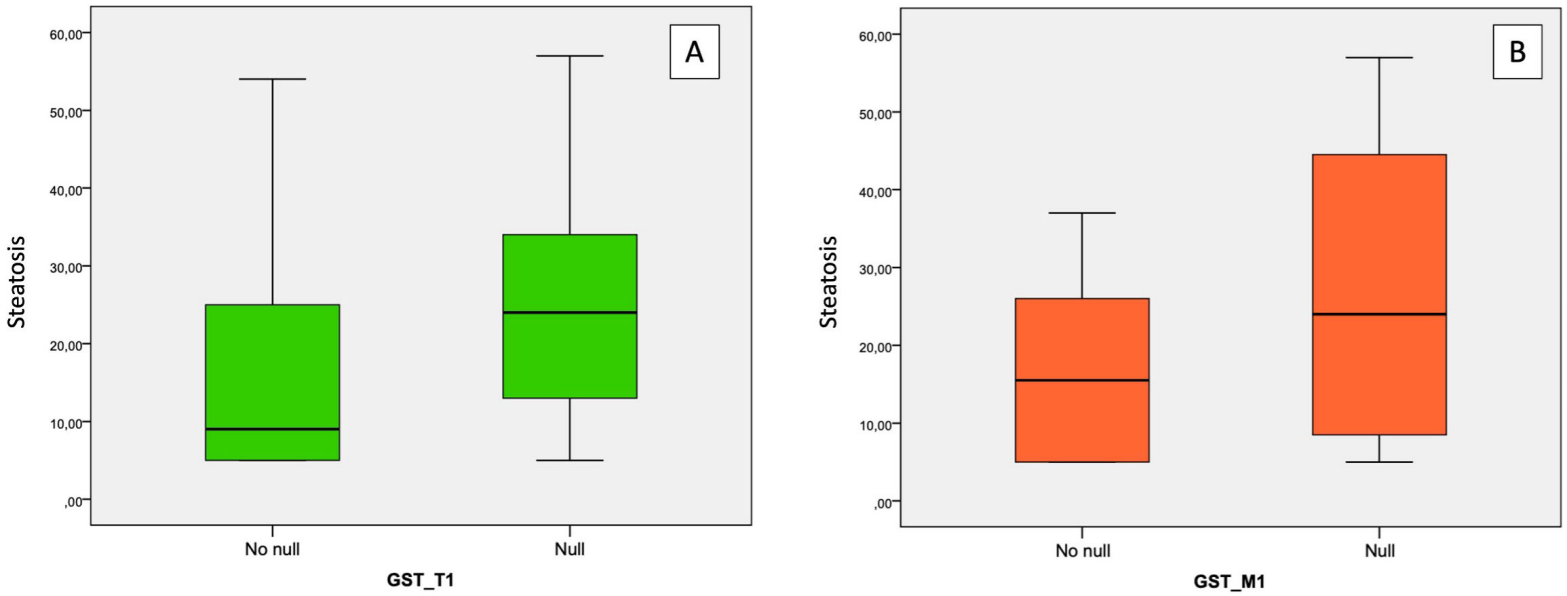
Association between “NULL” and “NO NULL” gene polymorphisms and hepatic steatosis grade. **(A)** correlation between GST-T1 and steatosis grade; **(B)** correlation between GST-M1 and steatosis grade. GST-M1, Glutathione S-transferases Mu 1; GST-T1, Glutathione S-Transferase Theta 1.

Both *GST-T1* and *GST-M1* “NULL” genotypes show a statistically significant association with the presence of steatosis: for GST-T1, *r* = 0.258, *p* = 0.005; for GST-M1, *r* = 0.260, *p* = 0.005 (**Tables 3A, B**). A high statistically significant association between the *GST-M1* “NULL” genotype and a higher grade of steatosis (moderate steatosis, 34%–66%) (*r* = 0.362, *p* < 0.001) was found. Therefore, subjects with GST-M1 “NULL” have a greater probability of developing not only steatosis but also a higher steatosis grade. On the other hand, no statistically significant association between the presence of the “NULL” *GST-T1* genotype and the steatosis grade was found. In fact, the ordinal logistic regression model allowed to highlight that both the *GST-M1* “NULL” and the *GST-T1* “NULL” genotypes were associated with a greater probability of developing steatosis: in particular, the *GST-M1* “NULL” genotype was associated with a greater risk (*p* = <0.001) of developing a higher grade of steatosis than the *GST-T1* “NULL” genotype (*p* = 0.039) (**Figures 5A, B**; **Table 4**).

**Table 3A:** Association between grade and GST-T1 polymorphism.

			GST_T1		Total
			NO NULL	NULL	
Degree	No steatosis	Count %	16	12	28
			30.2%	18.8%	23.9%
	Mild steatosis	Count %	29	35	64
			54.7%	54.7%	54.7%
	Moderate steatosis	Count %	8	17	25
			15.1%	26.6%	21.4%
Total		Count %	53	64	117
			100.0%	100.0%	100.0%
Pearson chi-square			3.370; *p* = 0.185		

**Table 3B:** Association between degree and GST-M1 polymorphism.

			GST_M1		Total
			NO NULL	NULL	
Degree	No steatosis	Count %	18	10	28
			27.3%	19.6%	23.9%
	Mild	Count %	46	18	64
			69.7%	35.3%	54.7%
	Moderate	Count %	2	23	25
			3.0%	45.1%	21.4%
Total		Count %	66	51	117
			100.0%	100.0%	100,0%
Pearson chi-square			30.758; *p* < 0.001		

**Table 4:** Results of the ordinal logistic regression model for steatosis grade.

		Estimate	95% CI	S.E.	*P*-value
Threshold	Constant1	−0.138	−2.677; 0.400	1.295	0.915
	Constant2	2.690	0.090; 5.291	1.327	0.043
Position	Age	0.007	−0.039; 0.053	0.024	0.766
	Gender	−0.110	−0.828; 0.608	0.366	0.764
	GST_T1	1.526	0.721; 2.267	0.378	**0.039**
	GST_M1	1.568	0.761; 2.376	0.412	**0.001**

The values reported in bold are those statistically significant (p <0.05).

Age appeared inversely related to the *GST-T1* “NULL” genotype (*r* = −0.216, *p* = 0.019) (**Table 3B**); therefore, the risk of developing hepatic steatosis in early age was higher in subjects with the *GST-T1* “NULL” genotype (*p* = 0.019) than in those with the *GST-M1* “NULL” genotype (*p* = 0.073), leading to consider it as a risk factor for the onset of NAFLD. No statistically significant correlations between the “NULL” genotype and sex were detected.

The results are to be considered expected given that the trend of the “NULL” polymorphism does not present any evidence of gender in the relevant literature. The particular distribution of the enzymatic polymorphism is a geographical one.

## 4 Discussion

NAFLD is considered a complex disease resulting from the interactions between the environment and the different genetic profiles of each individual, which makes them susceptible to develop the disease and to influence its progression. In recent years, we have witnessed multiple genome-wide associations, which have then enriched our knowledge on the genetic basis of NAFLD (6, 20, 21).

Furthermore, more recently, direct evaluation of hepatic fat fraction by MRI led to an estimated heritability of NAFLD in the general population at approximately 52% (22, 23).

In the conducted study, 117 healthy subjects with no risk factors and appropriately selected from the general population using specific questionnaires were enrolled. Genetic analysis was carried out to identify the presence of *GST-T1* and *GST-M1* “NULL” and “NO NULL” polymorphisms. Subsequent MRI examination to evaluate the grade of steatosis was performed. These analyses were performed to evaluate any correlation between the aforementioned gene polymorphism and NAFLD.

In recent years, in the literature, increasing importance in the possible pathogenesis of NAFLD is attributed to the loss of function of some genes, including those encoding for both phase I and phase II metabolic enzymes, as *GST* families (24–26). In this perspective, oxidative stress is considered to play a key role in determining liver injury, leading to cell damage and then progression in NAFLD (27). Oxidative stress is due to the production of ROS during metabolic processes: ROS accumulation is usually prevented by the action of antioxidant systems; therefore, their malfunction can lead to its progressive increase (28, 29). At high concentrations, ROS can determine oxidative modifications of cellular macromolecules, such as lipids, resulting in their accumulation into hepatocytes. ROS promotes lipid peroxidation, causing the formation of extremely reactive aldehyde compounds and damage at the intracellular level (30). Thus, one of the mechanisms by which ROS could lead to NAFLD onset and progression may be related to indiscriminate oxidative biomolecular damage although specific molecular pathways are not yet clearly defined (31).

Despite that, various other mechanisms have been reported to cause lipid peroxidation: pro-oxidant systems, such as cytochrome P450, lipoxygenase, and cyclooxygenase, have been solely or synergistically implicated in the emergence of OxS in NAFLD (26).

Since *GST* enzymes intervene in ROS catabolism and detoxification processes, a reduction in their functionality can determine their increase, potentially predisposing to the onset of NAFLD (26).

MRI is considered a non-invasive method for the evaluation of intrahepatic fat accumulation and then steatosis grade (32, 33).

Steatosis is typically graded on a 0–3 scale based on the number of cells with intracellular vacuoles of fat: grade 0 (normal) = up to 5% of cells affected; grade 1 (mild) = 5% to 33% of cells affected; grade 2 (moderate) = 34% to 66% of cells affected; and grade 3 (severe) = 67% or greater of cells affected (34). MRI exploits the difference in the resonance frequencies between water and fat proton signals. By acquiring the images at echo times in which water and fat signals are approximately in-phase (*W*+*F*) and opposed phase (*W*−*F*), volumetric liver fat detection is possible according to the relative signal loss on opposed-phase (also known as “out-of-phase”) images (35). Echo times for in-phase and opposed-phase imaging are based on the relative chemical shift between water and the methylene peak (–CH_2_) of fat (35). At 1.5 T and normal body temperature, this peak resonates approximately at −217 Hz, being slower than water (−434 Hz at 3.0 T) (35).

Unlike CT, where the pixel value directly reflects X-ray attenuation (in Hounsfield units), the signal intensity in MR images is arbitrary and depends on the receiver gain and the sensitivity of received RF coils. Fat signal fraction (*η*) can be calculated as


$$\eta =\frac{F}{W+F}$$

where *W* and *F* are the signal contributions from water and fat (35).

An attractive approach to create a PDFF map is to use a chemical shift based on the “water–fat separation method” that allows to separate the signal of both water and fat into water-only and fat-only images (36). Both the accurate separation of the signal of fat mobile protons from other mobile protons (i.e., water) and the correction for all those factors that influence MR signal intensity permit the calculation of PDFF: PDFF is defined as the density of hydrogen protons attributable to fat. To provide an accurate estimation of PDFF, the following five confounders must be evaluated: −T1 bias, −T2* decay, −spectral complexity of fat, −noise bias, and −eddy currents (36). After running the fix for the above five confounding factors, the fat-signal fraction and the PDFF are equivalent.

The right fat quantification techniques can be distinguished into two categories:

- “Magnitude-based”: This technique is easier to implement and uses two or more (six echo times) in-phase and opposed-phase images or magnitude images (18, 37); this method provides an estimate of the fat fraction, with a dynamic range of 0%–50% fat signal, which is probably sufficient to estimate the range of liver fat concentrations clinically encountered although it may not apply to all patients.
- “Complex-based”: This technique uses both magnitude and phase information from three or more images (37); this method provides estimates of fat fraction with a dynamic range of 0%–100% (38).

Although it is not possible to identify a threshold that allows to distinguish normal conditions from pathological ones, today, a reference threshold value of the fat fraction of 5.56% is commonly used (39).

The use of MRI as a quantitative biomarker of intracellular liver fat has shown tremendous progress in recent years and holds great promise to provide a cost-effective, accessible, and accurate evaluation of such a widespread disease (40, 41). Furthermore, MRI allows the acquisition of data comparable to those of conventional biopsy; however, it enables the entire liver parenchyma to be visualized, whereas only a small part is taken for histological examination.

In this study, MRI-PDFF has been used to detect the presence of hepatic steatosis in healthy subjects, apparently without any risk factors. Genetic analyses were conducted to identify “NULL” mutations of the *GST-T1* and *GST-M1* genes, evaluating their possible role in the genesis of NAFLD. As mentioned, these enzymes intervene in the catabolism processes of ROS; therefore, their loss of function (“NULL” genotype) is able to determine an increase in oxidative stress: ROS has been reported to cause lipid peroxidation, predisposing to the onset of NAFLD itself (41).

Four groups of cytological *GSTs* have been identified in humans, classified with the Greek letters alpha (α), mu (µ), pi (π), and theta (θ), and defined based on their isoelectric point (40). Four gene sequences were investigated for these enzymes: *GSTA* (*GSTα*), located on chromosome 6; *GSTM* (*GSTµ*), located on chromosome 1; *GSTP* (*GSTπ*), located on chromosome 11; and *GSTT* (*GSTθ*), located on chromosome 22. Several studies have described the specific structure, enzymology, and affinity of the tissue and gender of *GST-M* and *GST-T* polymorphisms (42). The enzymatic chains encoded by *GST-M1* polymorphism are expressed by hepatic, gastric, and nervous systems, while the enzymatic chains encoded by *GST-M2* and *GST-M5* polymorphisms are expressed by extrahepatic tissues and synthesized by other cell lines (43).

The genetic polymorphisms *GST-T1* and *GST-T2* are located in the same region on chromosome 22, and the related enzyme chains are mainly expressed in the liver (44).

The genetic polymorphism *GST-M1* can be typed by highlighting three allelic forms: GST-M1*0, GST-M1*A, and GST-M1*B. The GST-M1*0 allele (homozygous null) indicates a total deletion of the gene tract with a consequent lack of expression, at the cellular level, of the related enzyme chains (44). Alleles GST-M1*A and GST-M1*B differ from each other for a single base at the level of exon 7 and encode for protein monomers that constitute the functionally active isoform of the enzyme. It is also important to highlight that approximately 50% of the Caucasian population does not express the enzyme in relation to the gene deletion which, in homozygosity, gives rise to the GST-M1*0/*0 genotype (45). Another significant deletion is expressed at the level of the *GST-T1* polymorphism; the null genotype of *GST-T1* is expressed in approximately 15% of the Caucasian population and in approximately 60% of the Chinese and Korean populations (46).

According to the role played by these enzymes in the biotransformation of endogenous and exogenous toxicants, including carcinogens (for example, polycyclic aromatic hydrocarbons, PAHs), pollutants, alcohol, drugs, and other xenobiotic agents, it is probable that they play an important role even on the onset of pathologies widely spread in the general population, such as NAFLD (45, 46). The main mechanism appears to be related to the increase in oxidative stress and its consequences (46).

The “NULL” *GST-T1* polymorphism has been associated with higher TNF-α concentration when compared with the “NO NULL” polymorphism; thus, it could indicate activation of the proinflammatory segment of the cytokine profile and inflammatory processes (47). Noteworthily, TNF-α is considered to play a pivotal role in those mechanisms leading to IR, inflammation, and apoptosis in the case of NAFLD; thus, its elevated level has been considered a predictive factor of NAFLD progression (48). Alterations in the adipokine profile were instead detected in subjects with the *GST-M1* “NULL” genotype: leptin plasma levels were significantly higher than in patients with the “NO NULL” genotype. The high leptin level could be related to a high TNF-α concentration, which is capable of stimulating leptin production (49).

Prysyazhnyuk et al. (50) also documented a reduction of restored glutathione in patients with NAFLD and *GST-T1* and *GST-M1* gene “NULL” polymorphisms. A higher level of reaction products of thiobarbituric acid has also been detected in subjects with the zero genotype of the *GST-M1* gene than in those with the functional allele of the gene. Furthermore, Kassab et al. (51) claimed that the “NULL” genotypes of *GST-T1* and *GST-M1* genes could determine an activity reduction of sulfhydryl binding and then a loss of detoxification body capacity. Despite this, some authors maintained that the presence of the “NULL” polymorphism alone cannot determine a relevant lack of *GST* isoenzyme synthesis to determine a greater susceptibility to genetic damage (52). In the literature, several studies have evaluated the different distribution of *GST-T1* and *GST-M1* polymorphisms between subjects with NAFLD and the rest of the general population. Hori et al. (53) conducted a study involving the Japanese population, reporting a higher frequency of the *GST-M1* null genotype in NAFLD patients as compared to the control group. Similar results were obtained in the study conducted by Kordi-Tamandani et al. (54). On the other hand, Hori et al. (53) found liver function alterations in the presence of “NULL” *GST-T1*, *GST-P1*, and *GST-M1* polymorphisms, with the synthesis, detoxification, and excretion parameters altered. No alterations in the lipid profile were found either (53). Different data have been obtained by Maciel et al. (55), who found hypertriglyceridemia in the presence of the aforementioned “NULL” polymorphisms. Furthermore, in agreement with the study of Hori et al., Prysyazhnyuk et al. (56) in a study conducted in Ukraine found a higher prevalence of “NULL” polymorphisms among NAFLD patients than in healthy individuals. Zhu et al. (40) conducted six studies including 700 NAFLD patients and 1,317 controls, aiming at identifying the frequency of *GST-M1* gene polymorphism in subjects with NAFLD: it was revealed that *GST-M1* is appreciably connected with NAFLD. Similar results have been obtained for *GST-T1* “NULL” polymorphism distribution: five studies involving 620 NAFLD and 1,237 healthy subjects found a noticeable association between the SNP of *GST-T1* and NAFLD vulnerability. Therefore, these data suggest a significant correlation between *GST-M1/T1* “NULL” polymorphisms and the onset of NAFLD. These data are in agreement with the results of our study. Both *GST-M1* and *GST-T1* showed a higher prevalence in subjects with NAFLD although *GST-M1* showed a greater strength of association. Furthermore, the *GST-M1* NULL genotype is related to the onset of a higher grade of steatosis. Damavandi et al. (12) analyzed 242 NAFLD patients and 324 healthy controls in the Iranian population, reporting a higher prevalence of the *GST-M1* and *GST-T1* “NULL” genotypes in the first group. Subjects with those polymorphisms were then considered at a higher risk of developing NAFLD. Another study conducted by Hashemi et al. (6) in the Iranian population on 83 patients with NAFLD and 93 healthy subjects confirmed an increased risk of developing NAFLD in subjects with the *GST-M1* “NULL” genotype. On the other hand, the *GST-T1* “NULL” polymorphism distribution was not significantly different between NAFLD and control groups. Therefore, this study showed that *GST-M1*, but not *GST-T1*, can be considered a risk factor for developing NAFLD. These data disagree with what has been obtained in our study: in fact, although a stronger statistical association was identified between NAFLD and “NULL” *GST-M1* polymorphism, also the “NULL” *GST-T1* genotype has a statistically significant correlation. Therefore, it could also be considered an NAFLD genetic predisposing factor. The main data are summarized in **Table 5**.

**Table 5:** Main data of the analyzed articles.

Authors	Year	Country	Ethnicity	GST polymorphisms analyzed	Grade of steatosis detected	Sex prevalence	Statistical significance
Hashemi (6)	2012	Iran	Asian	GST-M1, GST-T1, and GST-P1	Not specified	Both sexes involved	Significant for GST-M1; GST-P1 considered as risk factor
Damavandi (12)	2021	Iran	Asian	GST-M1 and GST-T1	Mild to severe	Both sexes involved	Significant for GST-M1 and GST-M1 (high risk)
Zhu (40)	2022	Not Applicable	Not Applicable	GST-M1, GST-T1 and GST-P1	Not specified	Both sexes involved	Significant for both GST-T1 and GST-M1
Prysyazhnyuk (50)	2015	Ukraine	Caucasian	GST-M1 and GST-T1	Not specified	Both sexes involved	Significant for both GST-M1 and GST-T1
Hori (53)	2009	Japan	Asian	GST-M1, GST-T1 and GST-P1	Mild to severe	Both sexes involved	Significant for GST-M1, GST-M1 and GST-P1 (Ile105Va)
Kordi-Tamandani (54)	2011	Iran	Asian	GST-T1 and GST-P1 (methylation)	Not specified	Both sexes involved	Not significant
Maciel (55)	2009	Brazil	Not reported	GST-M1, GST-T1 and GST-P1	Not specified	Both sexes involved	Significant for GST-M1, GST-M1 and GST-P1 (increased risk of hypertriglyceridemia)
Prysyazhnyuk (56)	2017	Ukraine	Caucasian	GST-P1	Mild to severe	Both sexes involved	Significant

Furthermore, the correlation between GST-T1 and GST-M1 “NULL” polymorphisms and the etiopathogenesis of hepatic steatosis can be considered a challenge for forensic genetic laboratories since screening assumes the significance of biomarker of *individual susceptibility* in genetic predispositions to metabolic diseases especially in those subjects professionally exposed to xenobiotic substances. Genetic analysis could help us to understand some pathological findings in forensic cases in ascertaining the cause of steatosis even in the absence of specific dietary and/or environmental risk factors. Further forensic applications include the study of polymorphisms in association with liver function for those cases in which it is necessary to interpret the degree of drug biotransformation in *postmortem toxicological investigations* as a contribution to *molecular autopsy*. Therefore, the identification of genetic biomarkers linked to hepatic steatosis can support more accurate forensic and toxicological evaluations. The biomolecular interest in identifying the causes of hepatic steatosis cannot be overlooked, as this is a pathology of significant social interest for the resulting social security and tertiary protection aspects. Based on what has been described, the analysis of the correlation between the GST-M1 and GST-T1 NULL genotypes and hepatic steatosis would seem promising, and there are several future perspectives related to this area of research: i) the analysis of the role of both oxidative stress and lipid metabolism, ii) the interactions with environmental factors and other polymorphisms, iii) the potential role of GST NULL genotypes as risk factors and early biomarkers for the identification of hepatic steatosis, iv) the possibility of developing tailored prevention interventions based on genetic risk and thus identifying paths for the development of new strategies, and v) reconsidering social security and public protection strategies and the allocation of resources are certainly among the most relevant.

### 4.1 Limitations and risk of bias

Despite the analyses performed, some possible limitations of the study must be reported. Although the enrolled subjects have a BMI within the limits of normality and declare they are not affected by any pathology, the blood analyses only allow to exclude dysmetabolic pathologies (diabetes mellitus, dyslipidemia, etc.), but it was not possible to ascertain the real absence of other pathologies. In addition, the information regarding eating habits generally refers to the number of meals during the day, the consumption of fruits and vegetables, the quantity of alcohol consumed daily, etc. Therefore, for instance, no specific information has been acquired on the medium daily caloric intake.

## 5 Conclusion

In conclusion, the study conducted by this research group aimed to demonstrate a correlation between the presence of the *GST-T1* and *GST-M1* “NULL” genotypes and the presence of steatosis. Thanks to the analysis of the collected data, it was possible to identify a statistically significant correlation between the presence of the *GST-T1* and *GST-M1* “NULL” genotypes and the presence of hepatic steatosis, which is higher for *GST-M1* “NULL.” Furthermore, a high statistically significant association between the *GST-M1* “NULL” genotype and a higher grade of steatosis was found. In contrast, no statistically significant association between the *GST-T1* “NULL” genotype and steatosis grade was identified. It was also possible to underline the importance of MRI-estimated PDFF in the diagnosis of hepatic steatosis: this method allows the acquisition of data comparable to those of conventional biopsy; however, it permits the entire liver parenchyma to be visualized and only a small part is taken for histological examination. Despite this, considering both the stringent exclusion criteria that led to the healthy subjects without risk factors enrolled and the small number of the population analyzed, further studies are required to confirm the data obtained.

## Data Availability

The original contributions presented in the study are included in the article/supplementary material, further inquiries can be directed to the corresponding authors.
